# Developmental Stability Covaries with Genome-Wide and Single-Locus Heterozygosity in House Sparrows

**DOI:** 10.1371/journal.pone.0021569

**Published:** 2011-07-01

**Authors:** Carl Vangestel, Joachim Mergeay, Deborah A. Dawson, Viki Vandomme, Luc Lens

**Affiliations:** 1 Terrestrial Ecology Unit, Department of Biology, Ghent University, Ghent, Belgium; 2 Research Group of Genetic Diversity, Research Institute for Nature and Forest, Geraardsbergen, Belgium; 3 Department of Animal and Plant Sciences, University of Sheffield, Sheffield, South Yorkshire, United Kingdom; University of Arkanas, United States of America

## Abstract

Fluctuating asymmetry (FA), a measure of developmental instability, has been hypothesized to increase with genetic stress. Despite numerous studies providing empirical evidence for associations between FA and genome-wide properties such as multi-locus heterozygosity, support for single-locus effects remains scant. Here we test if, and to what extent, FA co-varies with single- and multilocus markers of genetic diversity in house sparrow (*Passer domesticus*) populations along an urban gradient. In line with theoretical expectations, FA was inversely correlated with genetic diversity estimated at genome level. However, this relationship was largely driven by variation at a single key locus. Contrary to our expectations, relationships between FA and genetic diversity were not stronger in individuals from urban populations that experience higher nutritional stress. We conclude that loss of genetic diversity adversely affects developmental stability in *P. domesticus*, and more generally, that the molecular basis of developmental stability may involve complex interactions between local and genome-wide effects. Further study on the relative effects of single-locus and genome-wide effects on the developmental stability of populations with different genetic properties is therefore needed.

## Introduction

Developmental stability refers to the ability of an organism to achieve a phenotypic endpoint, predetermined by its genotype and the environment, along a developmental pathway in the face of random perturbations [1]. Because developmental stability has been shown to decrease with environmental and genetic stress and to correlate with fitness traits such as fecundity, attractiveness, competitive ability, parasite resistance and survival (see reviews in for example [1], [2], [3]), it has received much attention in ecology and conservation biology. Furthermore, as developmental instability may increase morphological variation and reveal cryptic genetic variation (e.g. [4]), it can affect evolutionary processes, and possibly speciation, too [5].

Population and individual levels of developmental stability are most commonly estimated by corresponding levels of fluctuating asymmetry (FA), i.e. small, random deviations from perfect left-right symmetry in bilateral traits [6]. Developmental stability and FA are inversely related to one another as high levels of FA reflect poor developmental stability. Developmental theory assumes that left and right trait sides reflect two independent replicates of the same developmental event and should therefore develop symmetrically in the absence of random perturbations [3]. While empirical studies revealed positive relationships between FA and genetic stress (reviewed by [7], [8]), numerous inconclusive examples nourish the debate over the generality of these relationships [9], [10]. Relationships between FA, stress and fitness have not always been consistent in the past but seem to be highly variable and species, stress and trait specific [2], [3]. Heterogeneity in the strength or direction of relationships with FA may result from complex genotype-environment interactions [11]. For example, the fact that relationships between FA and heterozygosity were only significant under suboptimal rearing conditions in the freshwater fish *Gambusia holbrooki* [12], suboptimal foraging conditions in the forest bird *Turdus helleri* [13], and suboptimal growing conditions in the flowering plant *Lychnis viscaria* [14], suggests that developmental stability may be traded-off against other vital life-history traits when individuals become energetically challenged [12], [13], [15].

Based on developmental and genetic theory, at least two hypothetical mechanisms underlying the genetic basis of developmental stability have been put forward [7], [9,]: (i) the *heterozygosity hypothesis* states that individuals with high levels of protein heterozygosity are developmentally stable as a result of dominance or overdominance effects [8], [16], [17], [18]. Genetic dominance refers to increased expression of deleterious recessive alleles in homozygote individuals [19], whereas genetic overdominance refers to superior biochemical efficiency of individuals that are heterozygous for genes at marker loci (‘true overdominance’) or at non-neutral genes tightly linked to the latter (‘associative overdominance’) [7], [20], [21], [22]. Both genetic dominance and ‘associative’ overdominance implies genetic disequilibria, however, the ecological conditions under which these disequilibria occur, can differ. Genetic dominance is most strongly associated with non-random association of diploid genotypes in zygotes (identity disequilibria) which is common under partial inbreeding [17]. Associative overdominance, in turn, is more strongly associated with non-random associations of alleles at different loci in gametes (linkage disequilibria), which typically occurs under recent population bottlenecks followed by rapid population expansion or intermixing of genetically differentiated populations; (ii) the *genomic co-adaptation hypothesis* states that balanced co-adapted gene complexes result in higher developmental stability because natural selection favours alleles at many different loci that ‘harmoniously’ interact during the developmental process to produce stable phenotypes [7], [9], [16]. Strong selection or outbreeding has been shown to break up such co-adapted gene complexes [7], [9].

While numerous studies have provided empirical evidence for associations between developmental stability and genome-wide processes such as multi-locus heterozygosity, evidence for single locus effects (local effect hypothesis *sensu* [16], [17], [23]) is still scant. A study of inactive/null alleles at lactate dehydrogenase (LDH) loci in rainbow trout (*Oncorhynchus mykiss*) showed reduced levels of developmental stability in heterozygotes, probably due to a reduction in enzyme activity despite potential beneficial effects of chromosomal heterozygosity [24]. A study on blowflies (*Lucilia cuprina*) showed that developmental stability in bristle numbers (but not wing characters) initially decreased upon exposure to a new pesticide but restored after modification of the genetic background through natural selection [25]. While loss of developmental stability was first explained by a disruption of co-adapted gene complexes, further study revealed direct effects of single resistance and modifier genes [26]. Recently, transcriptional knockdown techniques demonstrated the involvement of heath shock protein genes in the molecular control of developmental stability in *Drosophila melanogaster* and *Arabidopsis thaliana* [27], [28].

Here we study how developmental stability in a metric trait co-varies with indices of genome-wide and single-locus genetic diversity in microsatellite markers, within and among 26 house sparrow (*Passer domesticus*) populations along an urban gradient. Despite the wealth of analytic tools developed for non-coding neutral markers and their presumed suitability to test relationships with genetic diversity, few studies have applied such markers to model single- and multi-locus relationships with developmental stability [29], [30], [31]. Based on the following ecological and genetic evidence, relationships between developmental stability and genetic diversity are predicted to be stronger in more urbanized areas. First, urban house sparrows are more strongly, energetically challenged than suburban and rural individuals [32], [33]. A previous study confirmed that a similar stress gradient was apparent within our study area [34]. Second, urban populations are on average smaller than suburban and rural ones (C. Vangestel, unpublished data). Under reduced population sizes, variation in inbreeding, estimates of genome-wide diversity based on restricted numbers of markers [21], and statistical power to detect relationships with developmental stability, are expected to increase. Individual-level FA and genetic estimates of multi-locus diversity show high sampling variability. The former represents variances based on two data points (e.g. left and right) while the latter attempts to estimate genome-wide characteristics using only a limited number of markers. As such, both estimates may become very noisy and are therefore regarded as weak estimates of complex biological processes such as respectively developmental stability [35], [36] and genome-wide diversity [37]. Consequently, associations between both estimates can be expected to be low (see [38] for a general discussion) while joint analysis of average values between groups can still be done with reasonable accuracy as long as the number of sampled individuals is high. As the strength of relationships between developmental stability and genetic diversity may hence vary with the hierarchical level of statistical analysis [31], [39], [40], hypotheses are tested at the level of populations and individuals.

## Materials and Methods

### Ethics Statement

All procedures involving animals were reviewed and approved by the Animal Ethics Committee of Ghent University (Permit Number ECP 08/05).

### Study site

**Figure 1:**
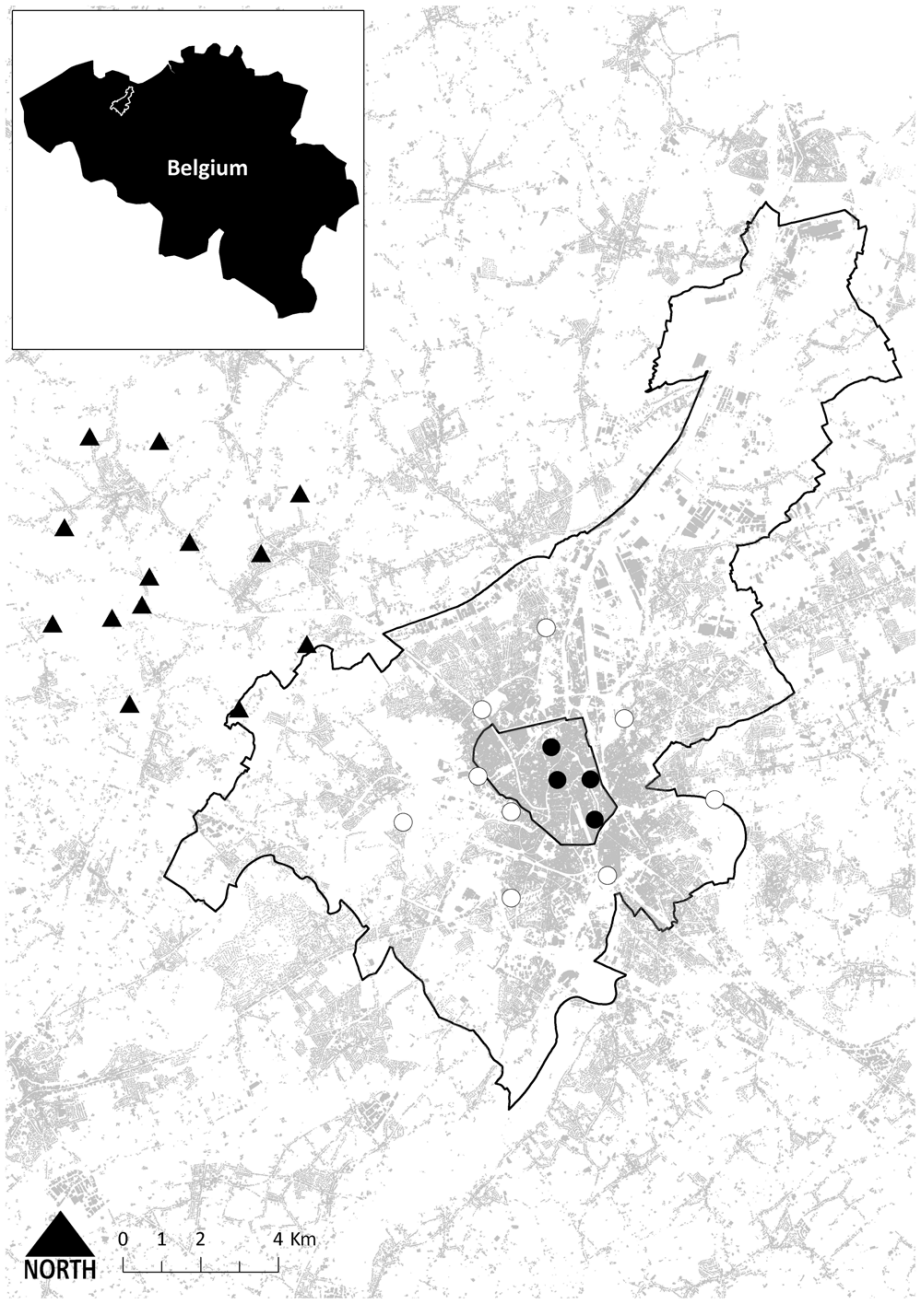
Geographical location of urban (filled circles), suburban (open circles) and rural (filled triangles) study plots within and near the city of Ghent (Belgium). Inner contour encompasses Ghent city centre, outer contour encompasses surrounding municipalities and grey shading represents built-up area.

House sparrows were sampled along an urban gradient ranging from the city centre of Ghent (northern Belgium) and its suburban periphery to the rural village of Zomergem, located ca. 12 km NW of Ghent. Urbanization was measured as the ratio of built-up to total grid cell area (each cell measuring 90,000 m^2^ on the ground) and ranged between 0–0.10 (‘rural’), 0.11–0.30 (‘suburban’) and larger than 0.30 (‘urban’) (Arcgis version 9.2.). We selected 26 plots along this gradient (Figure 1) in which we captured a total of 690 adult house sparrows by standard mist netting between 2003 and 2009 (equal sex ratios in majority of plots). Upon capture, each individual was sexed and aged and body mass (to the nearest 0.1 g), wing length (to the nearest 0.5 mm) and length of the left and right tarsus (to the nearest 0.01 mm; three repeated measurements sequenced left-right-left-right-left-right or vice versa and with digital slide calliper reset to zero between two consecutive measurements) were measured. Before release, we collected a small sample of body feathers for DNA analysis and the left and right fifth rectrix (counting outward) for feather growth analysis [34].

### Fluctuating asymmetry analysis

To estimate FA in tarsus length at an individual and population level, we carried out mixed regression analysis with restricted maximum likelihood parameter estimation (REML) to obtain unbiased individual FA estimates [41]. “Side” was modelled as a fixed effect, while “individual” and “individual*side” were modelled as random effects. Individual FA estimates were obtained from the individual random effects (“individual*side”). First, we modelled separate variances in measurement errors (ME) for each bird bander as the level of accuracy between banders might differ. Second we tested for the presence of directional asymmetry (fixed “side” effect; DA) by F-statistics, adjusting the denominator degrees of freedom by Satterthwaite's formula [42]. The distribution of signed FA in tarsus length showed a significant directional component in all bird banders as measurements of right tarsi were consistently larger than those of left ones. These differences were attributed to the specific handling of a bird when measuring both tarsi and therefore do not compromise the FA values as the mixed regression model corrects for this systemic bias by estimating subject-specific deviations from the fixed regression slope. Third, we tested the significance of FA by comparing the likelihood of models with and without random “individual*side” effect. Variation in length between repeated measurements within each side (ME) was significantly separated from variation between both trait sides (signed FA) (χ^2^ = 8454.7, d.f. = 1, p<0.001) and resulted in strong signal-to-noise ratios (all σ_FA_^2^/σ_ME_^2^ >9.4). Fourth, we calculated unbiased signed FA values (subject specific slope deviations from the fixed regression represented the amount of asymmetry after correcting for DA and ME). Finally, we calculated absolute values of the signed FA values (unsigned FA estimates, further referred to as “FA”) for hypothesis testing. These individual estimates were used for individual-based analyses while population mean values were used for analyses conducted at the population level.Fifth, we compared the kurtosis levels of the signed FA values to detect antisymmetry [43]. Visual inspection of signed FA values did not indicate the presence of antisymmetry as platycurtotic distributions were absent.

### DNA extraction, PCR and genotyping

Genomic DNA was extracted from ten plucked body feathers using a Chelex resin-based method (InstaGene Matrix, Bio-Rad) [44]. Polymerase chain reactions were organized in four multiplex-sets and included both traditional ‘anonymous’ microsatellites as well as those developed based on expressed sequence tags. For all loci full sequence length, chromosome location on the zebra finch genome and the nearest known zebra finch gene are given in an appendix (Table S1) (genome locations were assigned using WU-BLAST 2.0 software). The first multiplex reaction contained Pdoµ1 [45], Pdo32, Pdo47 [46] and TG04-012 [47]; the second one contained Pdoµ3 [45], Pdoµ5 [48], TG13-017 and TG07-022 [47]; the third multiplex reaction contained Pdo10 [48], Pdo16, Pdo19, Pdo22 [46] and TG01-040 [47]; the last set consisted of Pdo9 [48], TG01-148 and TG22-001 [47]. PCR reactions were performed on a 2720 Thermal Cycler (Applied Biosystems) in 9 µL volumes and contained approximately 3 µL genomic DNA, 3 µL QIAGEN Multiplex PCR Mastermix (QIAGEN) and 3 µL primermix (concentrations were 0.1 µM (Pdoµ1), 0.12 µM (TG01-148), 0.16 µM (Pdo10, Pdo19, Pdo22, Pdo32, TG04-012) and 0.2 µM (Pdoµ3, Pdoµ5, Pdo9, Pdo16, Pdo47, TG01-040, TG07-022, TG13-017, TG22-001)). The applied PCR profile used included an initial denaturation step of 15 min at 95°C, followed by 35 cycli of 30 s at 94°C, 90 s at 57°C and 60 s at 72°C; followed by an additional elongation step of 30 min at 60°C and an indefinite hold at 4°C. Prior to genotyping samples were quantified using a ND1000 spectrometer (Nanodrop technologies) and adjusted to a final concentration of 10 ng/µL. Negative and positive controls were employed during extraction and PCR to rule out contamination of reagents and ensure adequate primer aliquot working, respectively. PCR products were visualized on an ABI3730 Genetic Analyzer (Applied Biosystems), an internal LIZ-600 size standard was applied to determine allele size, known standard samples were added to align different runs and fragments were scored using the software package GENEMAPPER 4.0. Only individuals for which at least 10 markers successfully amplified were selected for subsequent analyses.

### Genetic data analysis

Because the genotyping of noninvasive DNA samples is potentially prone to artefacts [49], [50] we tested for scoring errors due to stuttering or differential amplification of size-variant alleles that may cause drop-out of large alleles using MICRO-CHECKER [51]. The same program was also used to assess the observed and expected frequency of null alleles by comparing frequencies of observed and Monte Carlo simulated homozygotes [52]. All microsatellite loci (n = 16) were checked for Hardy-Weinberg and linkage equilibrium with GENEPOP 4.0 [53], [54]. Mean unbiased expected heterozygosity across all populations (H_e_ [55]) was computed for each locus using FSTAT 2.9.3.2. [56]. Individual genetic diversity was estimated by (i) standardized multilocus heterozygosity (hereafter called MLH [57]), (ii) Ritland inbreeding coefficients (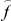, [58]) and (iii) squared differences in allele size (d^2^, [59], [60]).

**Figure 2:**
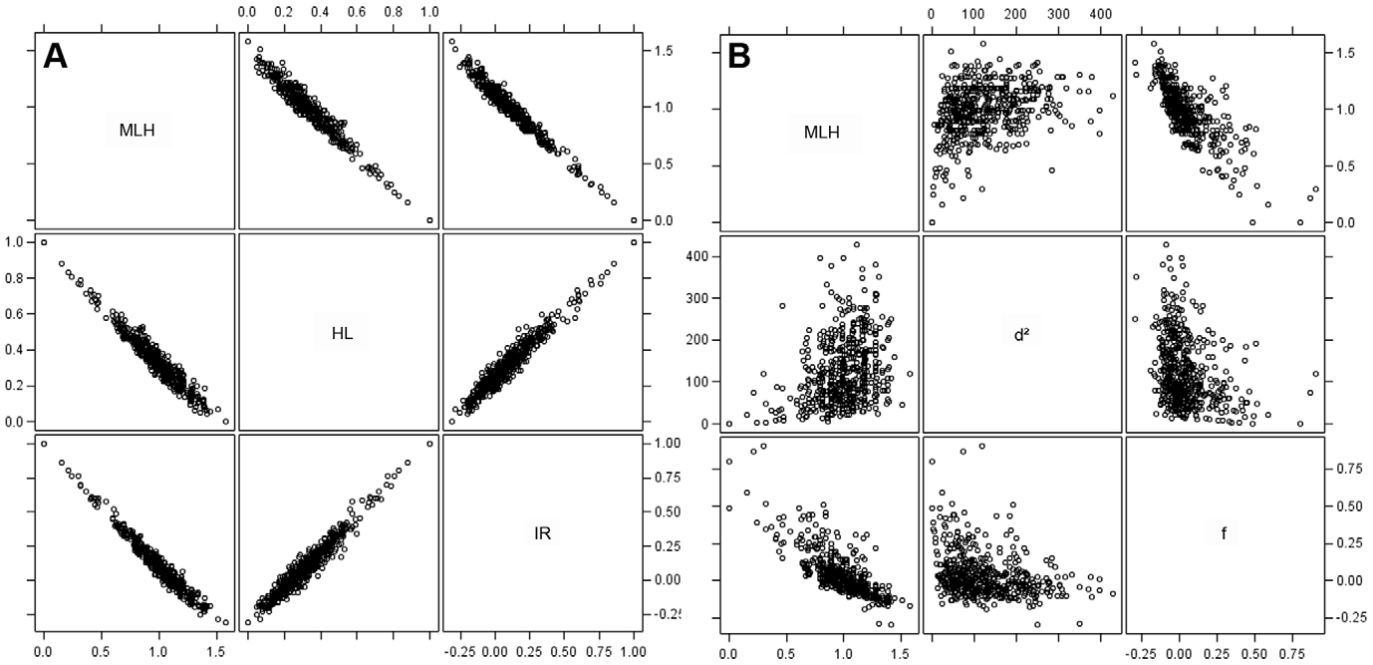
Correlation matrix between multi-locus (a) heterozygosity-based indices (MLH, HL and IR; see text for details) and (b) genetic diversity indices (MLH, d^2^ and 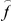).

- MLH was calculated as the ratio of the proportion of typed loci for which a given individual was heterozygote over the mean heterozygosity of those loci [57], thereby eliminating possible confounding effects of unbalanced datasets. Individual MLH estimates were calculated using Rhh [61], an extension package of R (http://www.r-project.org), which also provides two additional heterozygosity-based indices, i.e. homozygosity by loci (HL [62]) and internal relatedness (IR [63]). HL weighs the contribution of each locus and is calculated as HL  =  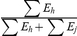, where E_h_ and E_j_ represent the expected heterozygosities of the homozygous and heterozygous loci, respectively. IR on the other hand incorporates allele frequencies to estimate levels of homozygosity. IR  =  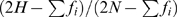, where H represents the number of homozygous loci, N the total number of loci and f_i_ the frequency of the *i*th allele in the genotype. Positive values reflect high levels of homozygosity while negative values are indicative for high heterozygosity. As all three indices were strongly correlated (all |r|>0.97; p<0.001) (Figure 2a) and results remained unaffected when based on MLH, IR or HL (despite differences in the relative weight given to alleles or loci when estimating heterozygosity [63]), only results of analyses with MLH are reported.
- Ritland estimates were obtained from the software program MARK (available at http://genetics.forestry.ubc.ca/ritland/programs.html) and calculated as 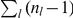/ 
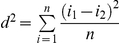
, where *i* and *l* represent alleles and loci, respectively; *S_il_* equals 1 if both alleles are allele *i* or 0 otherwise, *P_il_* is the frequency of allele *i* at locus *l* and *n_l_* denotes the number of alleles at locus *l* [58]. This unbiased method-of-moment estimator is thought to be particularly useful for highly variable markers since precision of the estimate is proportional to the number of alleles per locus [64].
- Mean squared distances between two alleles within an individual were calculated as mean 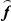, where *i_1_* and *i_2_* are the length in repeat units of allele *1* and allele *2* at locus *i* and n is the number of loci analyzed. By dividing all d^2^ values by the maximum observed value at that locus, effects of highly variable loci were accounted for [63], [65]. This estimator is thought to allow inference about the time since coalescence of two alleles, given that alleles of more similar length are more likely related by common ancestry [60], [65] and has proven to be a valuable measure in the event of recent admixture of highly differentiated populations and superior fitness of hybrid descendant due to heterosis. Under such conditions, d^2^ is hypothesized to be the most optimal fitness predictor asit integrates the migration signature into its estimate [65], unlike the other heterozygosity-based indices.

To test whether the number of genetic markers used in our study was sufficient to make valid inferences on genome-wide heterozygosity, we divided our marker set in two random subsets and calculated individual multilocus heterozygosity indices for each subset with the program Rhh [61]. In order to make the claim of genome-wide heterozygosity tenable, both subsets should yield comparable estimates of individual multilocus heterozygosity. Hence, individual multilocus heterozygosity estimates should be positively correlated and this procedure was repeated 1000 times to obtain confidence intervals for mean heterozygosity-heterozygosity correlations.

### Statistical analysis

We used Pearson correlation coefficients to quantify the strength of associations between d^2^, MLH and 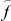, and general linear models with Gaussian error structure to study between-plot variation in genetic diversity and relationships with tarsus FA. Observer was added as a covariate to account for possible confounding effects of between-observer heterogeneity and analyses were tested at two hierarchical levels: among individuals and among populations (using mean values). Individual-based analyses were conducted in two ways. First, associations between FA and genetic diversity were estimated for each population using an ANCOVA model (population, genetic diversity and their interaction were modelled as fixed factors). An average within-population effect was estimated using a contrast statement. Second, individuals were pooled across all populations. While in the former model differences between populations are ignored, results from the latter model should resemble those of the population-level analysis if strong population effects are present. Initially, models were run for all markers combined (genome-wide effects). Next, the procedure was repeated per locus (unstandardized heterozygosity and d^2^ estimates) and the relationship between genetic variability and strength, measured as total variance explained, of these (single-locus) genotype-FA associations was assessed. Positive Spearman rank correlation coefficients imply that more heterozygous markers are more informative [23]. Finally, we applied a general linear mixed model to test whether associations between FA and genetic diversity varied with urbanization. Genetic diversity, urbanization, their interaction and bird bander were included as fixed factors Study plot and the interaction with each index of genetic variation were modelled as random effects. Degrees of freedom were estimated by Satterthwaite formulas to account for statistical dependence [66]. Per multi-locus genetic diversity index a sequential Bonferroni correction [67] was applied to account for multiple comparisons. All statistical analyses were performed with program SAS (version 9.2., SAS Institute 2008, Cary, NC, USA).

## Results

### Genetic diversity

All loci were highly polymorphic and most locus by population combinations were in Hardy-Weinberg equilibrium, yet some deviations reached significance after Bonferroni correction (three populations for Pdo47, two populations for Pdoµ5 and one for respectively Pdo9, Pdo32 and TG13-017). There was no evidence that scoring errors due to large allele drop-out or stutter contributed to this nonequilibrium. To ascertain these deviations did not influence our results we ran all analyses with and without these five markers. Removing these loci did not alter any of the overall conclusions, hence only results based on the total dataset are reported. There was no evidence for linkage disequilibrium between any pair of loci. Standard statistics for each marker are presented in Table 1. Estimates of genetic diversity were significantly correlated at the individual level, most strongly between 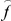 and MLH (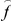 -MLH: r = −0.77, p<0.001; d^2^-MLH: r = 0.52, p<0.001; d^2^-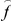: r = −0.40, p<0.001) (Figure 2b). MLH was a weak predictor of genome-wide heterozygosity as independent random sets of loci resulted in a low (but significant) positive heterozygosity-heterozygosity correlations (mean r =  0.16, 95% CI =  [0.10–0.22]). MLH and 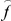 significantly varied among populations (resp. F_25,502_ = 2.35, p = 0.0003 and F_25,502_ = 2.00, p = 0.0031) while d^2^ showed a near-significant trend (F_25,502_ = 1.52, p = 0.053).

**Table 1:** Locus specific descriptive statistics for 16 microsatellite markers.

Locus	N	N_A_	H_o_	H_e_	f_null_
TG01-040	537	6	0.40	0.44	0.028 (0.01)
TG01-148	486	3	0.42	0.38	−0.023 (0.02)
TG04-012	549	5	0.53	0.59	0.039 (0.016)
TG07-022	493	5	0.37	0.41	0.026 (0.012)
TG13-017	547	8	0.52	0.64	0.074 (0.015)
TG22-001	478	11	0.34	0.41	0.054 (0.013)
Pdoµ1	550	20	0.80	0.85	0.024 (0.009)
Pdoµ3	515	19	0.83	0.85	0.015 (0.007)
Pdoµ5	523	22	0.76	0.82	0.033 (0.011)
Pdo9	442	31	0.65	0.75	0.052 (0.013)
Pdo10	596	18	0.78	0.82	0.021 (0.011)
Pdo16	549	17	0.81	0.84	0.016 (0.01)
Pdo19	573	9	0.60	0.62	0.008 (0.011)
Pdo22	578	16	0.73	0.72	−0.005 (0.011)
Pdo32	491	20	0.59	0.75	0.093 (0.015)
Pdo47	562	17	0.68	0.83	0.078 (0.013)

Number of individuals genotyped (N), number of distinct alleles per locus (N_A_), observed (H_o_) and expected (H_e_) heterozygosity and null allele frequency (f_null_).

### Multi-locus association between FA and genetic diversity

**Figure 3:**
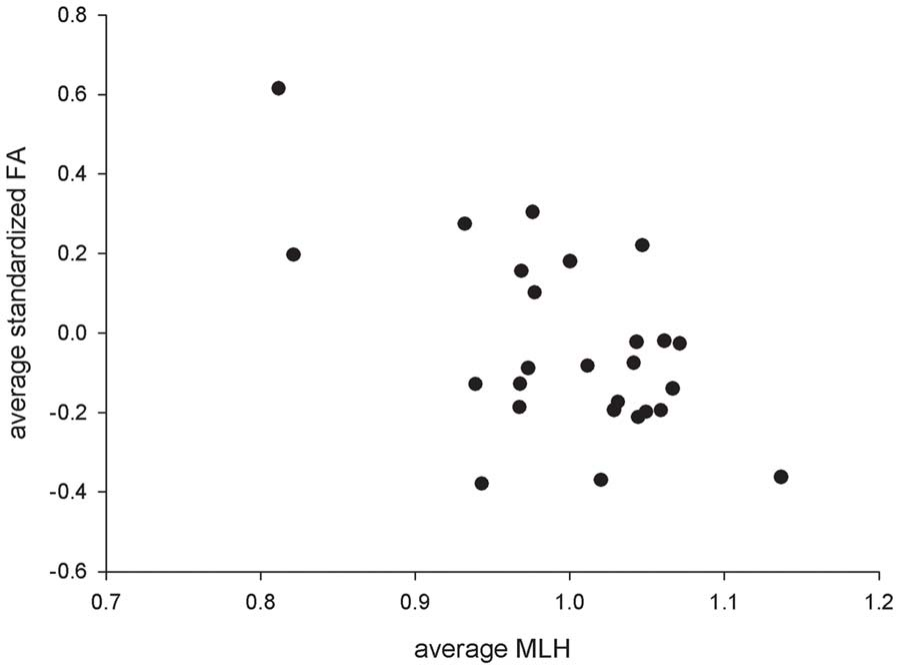
Inverse relationship between standardized multilocus heterozygosity and fluctuating asymmetry across 26 house sparrow populations.

Genetic diversity estimated by MLH and 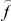 was significantly associated with tarsus FA modeled across all individuals. Highly homozygous individuals showed higher levels of FA compared to more heterozygous ones. However, MLH and 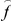 explained only little variation in FA (MLH: F_1,517_ = 6.59, p = 0.01, R^2^ = 0.049; 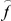: F_1,517_ = 4.70, p = 0.03, R^2^ = 0.046) (Table 2). When tested in each population separately, a similar (non-significant) trend occurred (MLH: F_1,467_ = 1.73, p = 0.19; 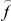: F_1, 467_ = 0.70, p = 0.40). As opposed to the weak associations measured at the individual level, mean values of MLH and 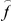 were strongly associated with mean levels of FA across all populations (MLH: F_1,24_ = 12.31, p = 0.001, R^2^ = 0.34; 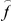: F_1,24_ = 7.88, p = 0.009, R^2^ = 0.25) (Figure 3; Table 2). In contrast, d^2^ was not correlated with FA at the individual nor population level (all p>0.15).

**Table 2:** Relationship between fluctuating asymmetry and three multi-locus genetic diversity estimates at three hierarchical levels of statistical analysis.

	d^2^					MLH					Ritland estimates				
	slope (SE)	F	num, den	p	R^2^	slope (SE)	F	num, den	p	R^2^	slope (SE)	F	num, den	p	R^2^
Individual level across all individuals	−1.20 (0.83)	2.11	1, 517	0.15	0.041	−0.49 (0.19)	6.59	1, 517	**0.01**	0.049	0.63 (0.29)	4.70	1, 517	**0.03**	0.046
Individual level within population	−1.02 (0.94)	1.18	1, 467	0.28	−	−0.30 (0.23)	1.73	1, 467	0.19	−	0.33 (0.39)	0.70	1, 467	0.40	−
Population level	−3.93 (3.12)	1.59	1, 24	0.22	0.062	−1.88 (0.54)	12.31	1, 24	**0.001**	0.339	2.18 (0.78)	7.88	1, 24	**0.009**	0.247

Significant tests are indicated in bold.

F = F-test, num,den =  numerator and denumerator degrees of freedom, R^2^ =  amount of variation in FA explained by heterozygosity.

### Single-locus association between FA and genetic diversity

**Figure 4:**
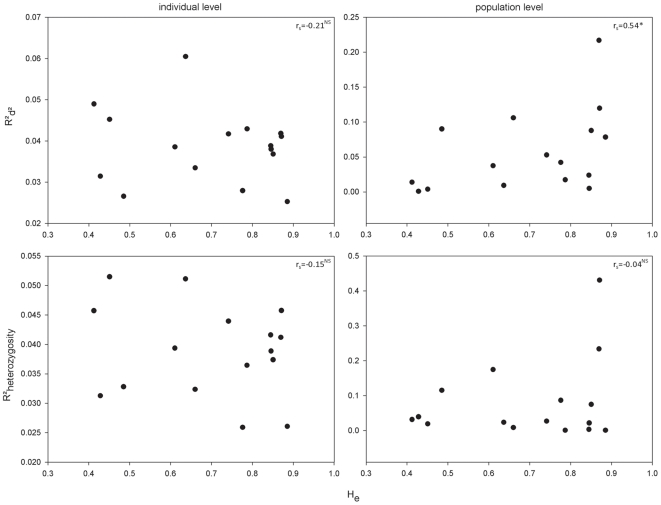
Relationship between locus specific variability (H_e_) and strength of the association between FA and genetic diversity. Left panes represent analyses at the individual level, right panes those at the population level. Associations are shown for two diversity indices: d^2^ (upper panes) and observed heterozygosity (lower panes).

Single-locus effects at the individual level were in concordance with those based on multiple loci, i.e. individual genotypes failed to explain variation in FA at each locus (all R^2^≤0.06). When analyzing each microsatellite locus separately, the association between heterozygosity and FA at population level was strongest at loci Pdoµ1, Pdo16 and TG04-012, whereas mean differences in allelic size were strongest at locus Pdo16. After sequential Bonferroni correction for multiple testing, the association at locus Pdoµ1 remained significant (Table 3). As all loci were in linkage equilibrium and heterozygosity-heterozygosity correlations were low, FA was modeled as a multiple regression with mean heterozygosity at each locus as independent variable. A model with all loci explained 85% of the variance in FA, whereas 43% of the variance was explained by a model with locus Pdoµ1 only, and 53% by a model with loci Pdoµ1 and Pdo16 only. After removing one or both loci, FA-MLH relationships remained significant (Pdoµ1 removed: F_1,24_ = 9.97, p = 0.0043, R^2^_MLH_ = 0.29 ; Pdoµ1+Pdo16 removed: F_1,24_ = 6.94, p = 0.015, R^2^_MLH_ = 0.22). The strength of single-locus FA-d^2^ relationships (16 loci) were positively correlated with expected heterozygosity at population level (r_s_ = 0.54, p = 0.03) but not at individual level (r_s_ = −0.21, p = 0.43). In contrast, FA-MLH relationships did not significantly vary with genetic diversity (all p>0.58) (Figure 4).

**Table 3:** Relationship between fluctuating asymmetry and single-locus genetic diversity at the individual (across all individuals) and population level.

		Individual level analysis								Population level analysis							
		d^2^				heterozygosity				d^2^				heterozygosity			
Locus	He	F	num, den	p	R^2^	F	num, den	p	R^2^	F	num, den	p	R^2^	F	num, den	p	R^2^
TG01-040	0.45	3.53	1, 495	0.06	0.045	6.8	1, 495	**0.01**	0.051	0.09	1, 24	0.76	0.004	0.47	1, 24	0.50	0.019
TG01-148	0.41	3.05	1, 374	0.08	0.049	1.77	1, 374	0.18	0.046	0.34	1, 24	0.57	0.014	0.78	1, 24	0.38	0.032
TG04-012	0.61	0.09	1, 474	0.77	0.039	0.48	1, 474	0.49	0.039	0.93	1, 24	0.34	0.037	5.09	1, 24	0.03	0.175
TG07-022	0.43	0.29	1, 433	0.59	0.031	0.21	1, 433	0.65	0.031	0.02	1, 24	0.90	0.001	0.99	1, 24	0.33	0.040
TG13-017	0.66	0.91	1, 469	0.34	0.033	0.38	1, 469	0.54	0.032	2.85	1, 24	0.10	0.106	0.21	1, 24	0.65	0.009
TG22-001	0.49	0.04	1, 369	0.84	0.027	2.4	1, 369	0.12	0.033	2.38	1, 24	0.14	0.090	3.13	1, 24	0.09	0.115
Pdoµ1	0.87	0.46	1, 495	0.50	0.041	2.86	1, 495	0.09	0.046	3.26	1, 24	0.08	0.120	18.17	1, 24	**0.001**	0.431
Pdoµ3	0.89	0.22	1, 400	0.64	0.025	0.55	1, 400	0.46	0.026	2.04	1, 24	0.17	0.078	0.02	1, 24	0.88	0.001
Pdoµ5	0.85	0.71	1, 442	0.40	0.038	1.1	1, 442	0.29	0.039	0.12	1, 24	0.73	0.005	0.53	1, 24	0.47	0.022
Pdo9	0.79	3.15	1, 378	0.08	0.043	0.62	1, 378	0.43	0.036	0.42	1, 24	0.52	0.017	0.01	1, 24	0.96	0.001
Pdo10	0.84	0.14	1, 477	0.71	0.039	1.51	1, 477	0.22	0.042	0.59	1, 24	0.45	0.024	0.08	1, 24	0.78	0.003
Pdo16	0.87	0.35	1, 482	0.56	0.042	0.02	1, 482	0.88	0.041	6.65	1, 24	**0.02**	0.217	7.33	1, 24	**0.01**	0.234
Pdo19	0.64	10.81	1, 481	**0.001**	0.060	6.01	1, 481	**0.01**	0.051	0.23	1, 24	0.64	0.009	0.58	1, 24	0.45	0.024
Pdo22	0.74	0.04	1, 500	0.84	0.042	1.19	1, 500	0.27	0.044	1.34	1, 24	0.26	0.053	0.67	1, 24	0.42	0.027
Pdo32	0.78	1.01	1, 446	0.31	0.028	0.08	1, 446	0.78	0.026	1.06	1, 24	0.31	0.042	2.28	1, 24	0.14	0.087
Pdo47	0.85	0.5	1, 476	0.48	0.037	0.78	1, 476	0.38	0.037	2.31	1, 24	0.14	0.088	1.95	1, 24	0.18	0.075

Statistical significance levels before (bold) and after (underlined) Bonferroni correction for multiple tests refer to a critical alpha-value of 0.05.

F = F-test, num,den =  numerator and denumerator degrees of freedom, R^2^ =  amount of variation in FA explained by heterozygosity.

### Effects of urbanization on FA- genotype relationships

The strength of FA-MLH relationships tested at the individual level significantly varied with urbanization (F_2,513_ = 4.25, p = 0.01): both variables were inversely related in rural populations (t_513_ = −3.73, p = 0.002), but unrelated in urban (t_513_ = −0.45, p = 0.65) and suburban (t_513_ = 0.95, p = 0.34) ones. In contrast, the strength and direction of FA- 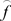 (F_2,41.6_ = 0.79, p = 0.46) and FA-d^2^ (F_2,507_ = 2.05, p = 0.13) relationships did not vary with urbanization.

## Discussion

Estimates of genetic diversity and developmental stability, averaged across individuals, significantly co-varied in the direction expected by developmental theory, whereas individual estimates were only weakly associated. Both genome-wide and locus-specific estimates of genetic diversity strongly correlated with developmental stability at the population level, and this correlation was mainly driven by genetic variation at two key loci only.

Whether relationships between developmental stability and genetic variability are driven by genome-wide heterozygosity or local effect of key loci, remains a topic of much debate [7], [9]. Relationships between proxies of developmental stability and genetic variability have typically been based on limited numbers of loci only, which were implicitly assumed to represent genome-wide properties. Such assumption, however, is only justified when repeated random subsets of markers give rise to strong heterozygosity-heterozygosity correlations [61], and this premise is often violated in randomly mating populations [61], [68], [69]. As levels of heterozygosity among markers within individuals were only moderately correlated in this study, our results do not fully support the role of genome-wide heterozygosity underlying relationships with developmental stability. Rather, single-locus effects at a few key loci, such as Pdoµ1, are more likely to drive these relationships.

Recent studies challenged the view that high levels of linkage disequilibrium are uncommon in natural populations, especially in small, bottlenecked or recently-mixed populations [70], [71], [72]. In addition, the selection of markers in genetic studies may be biased if based on the criterion of maximum variability [37], resulting in a slight overrepresentation of genes under balancing selection that retain enhanced levels of gene diversity due to heterosis. In our study, both markers that showed the strongest single-locus effects on developmental stability also displayed very high levels of heterozygosity. Likewise, fitness traits responded most strongly to the genetic constitution of the four most variable loci in a study on *Acrocephalus arundinaceus* [70]. Despite the fact that results from our study provide strong evidence for single-locus effects, genome-wide effects cannot entirely be ruled out as associations between FA and MLH persisted after removal of the two presumed key loci.

Unlike MLH and Ritland estimates, mean d^2^ only weakly predicted patterns in developmental stability at the population level. Results from this study hence support the conclusion that heterozygosity-based measures usually outperform those based on allelic distances like d^2^ to estimate inbreeding [73] and the negative appraisal of the use of squared distances between alleles to model relationships with fitness or its proxies [74]. Under recent admixture of genetically divergent populations [65], [75] or high variability at microsatellite loci [65], [74], [75], [76], however, the use of mean d^2^ may still be be justified. While some studies showed stronger genotype-fitness associations with increasing variability of the genetic marker under study [70], [77], others failed to detect such relationship [69] despite the theoretical prediction of such an effect [23]. Our results show that the effect of marker variability and relationships with proxies of fitness can be marker-dependent. The positive relationship in mean d^2^, but not in both other markers, may be explained by the fact that highly variable loci are thought to mutate in a step-wise mode, which is the underlying model for d^2^-based measures [65], [78], [79]. Yet, even at the most variable loci, mean d^2^ did not reach equal explanatory power compared to single-locus heterozygosity.

It has been hypothesized that the strength of relationships between developmental stability and genetic diversity may depend on other types of stressors [2] and that relationships with genetic stress or fitness may be more apparent under adverse conditions, i.e. when individuals are energetically challenged [12], [13], [15], [80]. Results of this study are not in concordance with this hypothesis since FA-heterozygosity associations were strongest in rural, not urban, populations. If juvenile mortality rates were higher in urban populations and selective in relation to FA, highly asymmetric and homozygous adults might be locally underrepresented, possibly changing the direction and/or strength of associations between FA and genetic variability. While nest studies on house sparrows revealed increased rates of nestling mortality when levels of insect abundance were critically low [33], levels of FA were not significantly lower in urban compared to suburban or rural populations in our study area [81] and observed proportions of homozygous individuals matched the expected ones as populations were in Hardy-Weinberg equilibrium. Hence, lack of support for interactive effects of nutritional stress and genetic diversity in the direction predicted, more likely resulted from low statistical power of individual-level analyses, although we cannot rule out that levels of stress during trait ontogeny in our study area were lower than those reported in the literature [33] as we did not quantitatively gauge the amount of perceived stress. Unfortunately, the restricted number of urban populations prevented us from testing the interactive stress hypothesis at the population level which would have assisted us in differentiating between low statistical power and an absence of stress as a possible explanation of the observed individual-level patterns.

In conclusion, results of this study provide strong evidence that relationships between developmental stability and heterozygosity can be driven by local effects at a few key loci, possibly in synergy with genome-wide effects, the relative contribution of which may depend on relative frequencies and fitness effects of deleterious genes [23]. Despite the fact that local linkage disequilibrium with key loci is regarded as the most promising mechanism to explain associations between developmental stability and heterozygosity, empirical support for the local effect hypothesis remains scant. Further research is therefore needed to unravel the relative effects of single-locus and genome-wide processes on developmental stability of populations with different genetic properties. While developmental stability earlier proved to be weakly associated with nutritional stress [81], relationships with heterozygosity appear stronger at the population level, irrespective of the underlying genetic basis. This study emphasizes again that the accuracy of developmental stability as a proxy for heterozygosity at the individual level remains low and the application of individual FA estimates in general should be abandoned.

## Supporting Information

Table S1 **Details of the 16 microsatellites used in this study, their location on the zebra finch (** ***Taeniopygia guttata*** **) genome and the position of the nearest known zebra finch gene.**
(PDF)
