# Supplementary material for: Developmental Stability Covaries with Genome-Wide and Single-Locus Heterozygosity in House Sparrows
Source: PLoS One. 2011 Jul 1;6(7):e21569. doi: 10.1371/journal.pone.0021569 (PMC3128584; doi:10.1371/journal.pone.0021569)
Supplement: Table S1 — Details of the 16 microsatellites used in this study, their location on the zebra finch ( Taeniopygia guttata ) genome and the position of the nearest known zebra finch gene. (PDF) [file pone.0021569.s001.pdf]

Table S1. Details of the 16 microsatellites used in this study, their location on the zebra finch (*Taeniopygia guttata*) genome and the position of the nearest known zebra finch gene.

Locus	EMBL accession number	Full sequence length	Location in the zebra finch genome[§]	ZF contig	Nearest gene on ZF map	Tgut name (Burt, Roslin)	Description gene
TG01-040[a,c]	DV576233	832	Chr 1A: 42,620,542	Contig20.15	Chr 1A: 42,621,707-42,624,796 reverse strand	DUSP6	Dual specificity protein phosphatase 6
TG01-148[a,c]	CK301512	849	Chr 1: 65,237,140	Contig3.570	Chr 1: 65,244,245-65,244,574 reverse strand	PDCH9-1	Protocadherin-9 precursor
TG04-012[a,c]	CK306810	657	Chr 4A: 17,044,573 (& unknown chr)	Contig15.1086	Chr 4A: 16,986,073-17,044,190 forward strand	ARHGEF9	Rho guanine nucleotide exchange factor 9
TG07-022[a,c]	DV948210	715	Chr 7: 11,940,140 & Chr7: 11,970,627	Contig5.1386	Chr 7: 11,915,850-11,964,575 reverse strand	IFIH1	Interferon-induced helicase C domain-containing protein 1
TG13-017[a,c]	CK313422	853	Chr 13: 18,542	Contig147.4	Chr 13: 18,142-21,075 reverse strand	EGR1	Early growth response protein 1
TG22-001[a,c]	CK317333	654	Chr 22: 1,428,098 (& unknown chr)	Contig117.57	Chr 22: 1,429,499-1,460,562 reverse strand	BNIP3L	BCL2/adenovirus E1B 19 kDa protein-interacting protein 3-like
Pdoμ1[b,d]	AM287188	191	Chr 1A: 34,300,915	Contig20.275	Chr 1A: 34,269,533-34,271,134 forward strand	DYRK2	Dual specificity tyrosine-phosphorylation-regulated kinase 2
Pdoμ3[b,d]	AM287190	277	Chr 8: 1,939,316 (& unknown chr)	Contig42.252	Chr 8: 1,951,927-2,011,591 reverse strand	PLA2G4A	Cytosolic phospholipase A2
Pdoμ5[b,d]	Y15126	390	Chr 4: 48,501,861	Contig11.688	Chr 4: 48,579,775-48,683,197 reverse strand	TBC1D1	TBC1 domain family member 1
Pdo9[b,d]	AF354423	518	Chr 24: 5,327,333 (& unknown chr)	Contig70.208	Chr 24: 5,258,154-5,276,865 forward strand	ATP21A	Potassium-transporting ATPase alpha chain 2
Pdo10[b,d]	AF354424	311	Chr 1: 58,669,562	Contig3.829	Chr 1: 58,299,864-58,682,577 forward strand	ENOX1	Ecto-NOX disulfide-thiol exchanger 1
Pdo16[b,d]	AM158995	294	Chr 2: 96,827,816	Contig34.193	Chr 2: 96,803,202-96,810,698 forward strand	ZADH1	Zinc-binding alcohol dehydrogenase domain-containing protein 2
Pdo19[b,d]	AM158998	341	Chr 2: 33,983,679	Contig4.1020	Chr 2: 33,910,198-34,156,663 reverse strand	not available	no description
Pdo22[b,d]	AM159001	146	Chr 4: 9,128,968	Contig92.145	Chr 4: 9,131,494-9,150,548 forward strand	IL15	Interleukin-15 precursor
Pdo32[b,d]	AM159011	361	Chr 1: 41,865,024	Contig8.931	Chr 1: 41,781,858-42,261,992 reverse strand	GPC6	Glypican-6 precursor
Pdo47[b,d]	AM159027	282	Chr5: 54,452,777	Contig1.2403	Chr 5: 54,573,873-54,712,474 reverse strand	BRF1	Transcription factor IIIB 90 kDa subunit

[a] EST based microsatellites, [b] Anonymous microsatellites, isolated via traditional cloning.

Source species: [c] zebra finch, [d] house sparrow.

[§] Genome locations in the zebra finch were assigned using the WU GSC BLAST software provided by the Washington University server following [1].

ZF, zebra finch *Taeniopygia guttata*.

References.

1. Dawson DA, Burke T, Hansson B, Pandhal J, Hale MC, et al. (2006) A predicted microsatellite map of the passerine genome based on chicken-passerine sequence similarity. Mol Ecol 15: 1299-1320.
